# Conspicuous Consumption in Emerging Markets: The Case of Starbucks in Colombia as a Global and Sustainable Brand

**DOI:** 10.3389/fpsyg.2021.662950

**Published:** 2021-08-19

**Authors:** Jose Andres Areiza-Padilla, Mario Andres Manzi Puertas

**Affiliations:** ^1^Department of Business Administration, Pontificia Universidad Javeriana, Bogotá, Colombia; ^2^Department of Marketing, University of Valencia, Valencia, Spain

**Keywords:** conspicuous consumption, global brands, sustainable brands, ethnocentrism, patriotism, brand image, brand loyalty, family allocentrism

## Abstract

Conspicuous consumption symbolizes ostentatious and material consumption through the purchase of products and services with a higher economic value and that allows their consumers to excel socially. However, conspicuous consumption often does not represent social and environmental values, because their products are often not characterized by being made through sustainable processes. United Nations, through its sustainable development agenda, invited companies to be aware of the social and environmental problems of the world and to be able to generate strategies that can be sustainable over time and allow the growth of both the company and society in general. This study analyses the case of Starbucks as a global and sustainable brand, which also generates conspicuous consumption in emerging markets. In this way, we explain how this brand generates a social status in emerging markets, which allow their consumers to excel socially. On the other hand, we explain how Starbucks contributes positively to sustainability in these types of markets, generating greater well-being for both the environment and society in general. In this way, this brand manages to transmit messages with symbolic values of social status, as well as social and environmental awareness. This research allows us to see how Starbucks is able to generate conspicuous consumption in Colombia, which is the third largest exporter of coffee in the world but is also capable of generating sustainable processes that help Colombian coffee growers and the environment. This research used a quantitative methodology based on a structured questionnaire made for conspicuous consumers of the Starbucks brand in Colombia, whose data were processed through the statistical program PLS 3.2.7. This research details the sustainable processes carried out by this brand in this developing country and the reasons why its consumption in this emerging market is considered conspicuous. For this research, family allocentrism, ethnocentrism, and patriotism are considered antecedents of conspicuous consumption, and brand image and brand loyalty are considered their results.

## Introduction

For Jung et al. (2020) conspicuous consumption is associated with values that allow showing a greater economic capacity through the products that are consumed in public, which generate to said consumers a reputation and a social status. However, the values of conspicuous consumption could be a barrier to sustainable consumption due to the eagerness to achieve ostentatious consumption at all costs. In this way, it is often pointed out that conspicuous consumption leads to social inequality and hinders sustainability, as this type of consumption attracts people who seek a clear social exposure through purchasing, the use and demonstration of products and services that allow them to excel socially; for this reason, one might think that global brands that generate a social status could promote these social inequities on a large scale, as they have presence in several countries of the world (Aliyev et al., 2019).

Jung et al. (2020) showed that the conspicuous consumer often associates sustainable products with products without design, little aesthetics, very outdated, and uninteresting; that is, with values that do not allow them to achieve a social distinction. However, this can change when brands have the ability to offer the conspicuous consumer products that are produced through sustainable processes but are, at the same time, eye-catching, original, and authentic. In this way, conspicuous consumers could use this type of sustainable products, which allow them to generate a social reputation.

For Aliyev et al. (2019), luxury companies can successfully adopt sustainability when they manage to improve communication and marketing processes. In this way, it seeks to know both the motivations that consumers have, to buy luxury products, as well as the motivations that they also have to buy sustainable products. In the same way, the same type of product could generate a double-purchase motivation. In this way, the idea is to be able to generate products that generate both symbolic and social satisfaction through sustainable luxury consumption. For Hardy and Van Vugt (2006), success in marketing products that are both luxurious and sustainable is based on being able to combine factors that generate both social altruism and factors that generate a status for the person who consumes them. In this way, such a consumer could demonstrate to the rest of society his or her desire for sustainable consumption but would also demonstrate his or her economic capacity above others.

This type of luxury and sustainable products would generate a benefit for the altruistic and economic reputation of the conspicuous consumer. In this way, if previously conspicuous consumption was perceived as a completely selfish consumption, nowadays, conspicuous consumption could be a strategy that allows promoting and exalting sustainable consumption practices. For this, celebrities or public figures who promote this type of consumption could be used, because these personalities exert a direct and indirect influence on other consumers (Griskevicius et al., 2010).

Considering this, in emerging markets, the purchase of certain products or brands becomes a reference toward an aspirational group of higher social status, in which the consumer is not currently, but would like to be included in the future. For this reason, in developing economies, the system of aspirational and ostentatious values is a very powerful force motivating conspicuous consumption. This is because there is a need to impress others through the consumption of certain products and brands, which symbolize economic progress (Huang and Wang, 2018). Due to the increased penetration of global brands in emerging markets, it is still necessary to carry out more studies to understand consumer behavior in these countries (He and Wang, 2017; Huang and Wang, 2018), and especially its relationship with conspicuous consumption due to the limited literature on these relationships (Aliyev et al., 2019; Anwar and El-Bassiouny, 2020; Jung et al., 2020).

With this in mind, there is now a need to generate marketing strategies that are in line with the sustainable development agenda proposed by the United Nations, in which companies must understand the impact of their decisions on society in general. In this way, various marketing strategies can be developed that allow social, environmental, and economic progress for both companies and society in general (Anwar and El-Bassiouny, 2020). For this reason, companies today must carry out processes in which the social impact of their marketing decisions is assessed, as, otherwise, their competitors could take a competitive advantage, anticipating various strategies for sustainable growth. It is important to mention that, today, the consumer is increasingly oriented toward sustainable consumption and, for this reason, usually prefers companies that also carry out sustainable practices (Epstein and Buhovac, 2017; Kotler et al., 2019). For this reason, some companies have successfully developed various strategies in all their processes in a sustainable way, which allow them to achieve a greater connection both with their consumers and with the society in which they operate (Peattie and Belz, 2010).

With this in mind, the main objective of this paper is to analyze some explanatory factors of conspicuous consumption in emerging markets. We wanted to analyze a global brand whose consumption generates a social status in emerging countries, while implementing a strategy that is in line with the sustainable development program proposed by the United Nations. In this way, this brand generates a commitment to the environment and to society in general.

This study focuses on the Starbucks brand and its development in an emerging country like Colombia, taking into account that Colombia is the third country exporting coffee in the world and where coffee is a symbol of national identity, with a strong consumption in this country. Through this study, we want to explain how a brand generates symbolic consumption of social status and, at the same time, symbolic consumption of social altruism.

Taking into account the above, this research paper presents the following structure: after presenting the introduction, we proceed with a review of the conspicuous consumption literature as the main variable and on the variables that we consider to be their antecedents and their results. For this study, family allocentrism, ethnocentrism, and patriotism were considered as antecedents of conspicuous consumption, while brand image and brand loyalty as their results. In this way, the relationships between the variables and their respective hypotheses are presented.

It is also explained why this research considers that the Starbucks brand generates conspicuous consumption in Colombia and the processes that it performs to generate sustainable production and marketing, with the environment and with the Colombian society. It then details the methodology that was used to verify the hypotheses, the results obtained, the conclusions, limitations, and future lines of research.

## Literature Review and Development of the Hypotheses

### Conspicuous Consumption

For Trigg (2001), theory of Thorstein Veblen of the idle class, written more than 100 years ago, now presents a powerful critique of the neoclassical theory of consumption since Veblen develops an evolutionary framework in which one can observe how preferences toward certain products determine certain social relations, with respect to the positions of individuals in the social hierarchy. In this way, according to theory of Veblen of conspicuous consumption, individuals often imitate the consumption behaviors of other individuals who possess a greater social position to try to resemble them. In this way, the higher the price, the more attractive the product, because the price becomes an indicator of prestige.

According to Belk (1988), an individual may become known to others, depending on the types of goods they may come to exhibit in front of others; in this way, the possessions of certain objects end up generating much importance for the one who has them and exhibits them, because these goods make it possible to communicate indirectly what a person can be; that is, we end up being judged according to the material goods we have and can show to others. Agreed with this, for conspicuous consumption of Grace and Griffin (2009) allows us to understand how the visible consumption of certain goods generates a better social position; in this way, individuals driven by this type of social values end up choosing products that allow them to convey the social image with which they wish to project themselves (Sheth et al., 1991). Given the above, for Braun and Wicklund (1989), the high price of products and the symbolism of status and prestige they represent in a given society are clear psychological antecedents of conspicuous consumption.

On the other hand, for Sirgy et al. (1991), the country of origin of a product can help to increase the desire to consume it, because the visibility of its consumption would also allow that social distinction in a certain population. That is to say that the good image that one has of the country of origin of this product would also be transmitted to who consumes it in a visible way for others, thus achieving public notoriety. For O'Cass and Frost (2002), one of the forces influencing consumer behavior is the desire for some to obtain social status through the purchase and consumption of some products and services, which allow them to have a clear social distinction. In this way, for some people, the purchase decision does not lie exclusively in the functional and rational features of the product you want to buy. In these cases, it also takes into account the symbolic characteristics that this product transmits, which allows you to meet your emotional needs through consumption of a visible form in front of the other people in your society (Al-Hyari et al., 2012).

In this way, in conspicuous consumption it is of vital importance to be able to generate a consumption that can be completely visible by other people. That is to say, direct observation by others is needed, through an open and public consumption, and not through a reserved consumption or without spectators (Piron, 2020). In this way, for Roy Chaudhuri et al. (2011), conspicuous consumption is a fully deliberate decision by a person who wants a symbolic possession or consumption that is visible to other people in order to achieve an image in front of others. In this way, the status generated by conspicuous consumption is presented through the consumption of certain brands, products, or services in a public way (Grace and Griffin, 2009).

For this research, we adopted the definition of conspicuous consumption of O'cass and McEwen (2004, p. 27): “conspicuous consumption focuses on the visual display or overt usage of products in the presence of others”; since we consider that the most important thing for the conspicuous consumer is to be able to show his or her consumption to other people. That is, it is not only valid to have the product but to be able to exhibit this consumption before society. In summary, we can find several approaches to the definition of this concept in the literature review in Table 1.

**Table 1:** Approximations to the conspicuous consumption.

**References**	**Definition**
Piron (2020, p. 309)	“Conspicuousness as the social and public visibility surrounding the consumption of a product. For consumption to be conspicuous, we expect it to be a social event, publicly witnessed by other consumers. Thus, publicly used products are more conspicuously consumed/used than products that are consumed/used in the privacy of one's home”
Grace and Griffin (2009, p. 15)	“Visible consumption of goods as a mechanism to enhance one's social standing”
Roy Chaudhuri et al. (2011, p. 217)	“Conspicuous consumption is a deliberate engagement in symbolic and visible purchase, possession and usage of products and services imbued with scarce economic and cultural capital with the motivation to communicate a distinctive self-image to others”
Assimos et al. (2019, p. 353)	“Conspicuous consumption should be seen as a way to increase prestige before society, through the public display of wealth”

*Source: Author's own compilation*.

### Family Allocentrism

Triandis and Gelfand (1998) proposed the terms “idiocentrism” by basing it on the characteristics of individualism and the term “allocentrism” by basing it on the characteristics of collectivism to distinguish the differences between these two social philosophies. Idiocentrics pay more attention to their own needs, and not so much to the needs of others; on the contrary, the allocentric people pay more attention to the needs of their group and not so much to their personal needs. For this reason, these types of people tend to prefer to fulfill goals of their groups first to their personal goals (Moon et al., 2018). Thus, for the allocentric, it is important to maintain good social relations (Dabul et al., 1995). Allocentric people emphasize family integration, where they tend to take into account the needs and concerns of members of their social group. Allocentrism is common in the regions of Asia, Africa, South America, and the Pacific; however, it is important to mention that there are several types of allocentrism according to specific cultural groups, such as family, friends, work, country, etc. (Triandis and Gelfand, 1998). For some authors, such as Li et al. (2018), this diversity of collective subgroups can generate ambiguity when trying to study them separately; for this reason, some researchers recommend that it be clarified specifically to which subgroup is referred.

Taking this into account, for Lay et al. (1998), family allocentrism specifically captures the allocentric orientations within the family context; it allows to identify the degree to which individuals are connected with their family. Individuals with a high level of family allocentrism tend to obey the expectations of family members, commit to family obligations, and prioritize family goals over personal goals (Li et al., 2018). In other words, family allocentrism makes it possible to identify the degree to which individuals are linked to their families. In this way, this buying behavior is influenced by the family and its family tradition (Gregory et al., 2002). In light of the above, for Wong and Ahuvia (1998), when a person makes a decision, he or she acts not only as a particular individual but as the representative of a particular group; and this behavior will also be reflected in the rest of this group. In this way, for Childers and Rao (1992), family allocentrism influences an individual in such a way that the consumption of certain products, services, or brands usually remains in time, passing from generation to generation as a symbol of family tradition.

Wong and Ahuvia (1998) conclude that, in allocentric-valued societies, conspicuous consumption not only reflects the social status of a particular individual but also reflects the social status of all the members of the family of the consumer. That is, if a person has the economic capacity to consume certain luxury products, it is understood that all the members of the family of this individual also have the economic capacity to buy luxurious products. Ger and Belk (1999) consider that in societies with allocentric tendencies, the wealth and positions of the family are a factor of social status that can feed conspicuous consumption. In this way, it can be understood why in collective societies and, especially, in those emerging countries the conspicuous consumption of family members is important, either as a way to stand out socially among the less favored population groups, or as a way to resemble an aspirational group of higher social status (Thoumrungroje, 2014; Huang and Wang, 2018).

Taking into account the previous studies, we formulated the following hypothesis in developing countries:

#### Hypothesis 1

Family allocentrism has a positive impact on the conspicuous consumption of global brands that practice sustainability in developing countries.

### Ethnocentrism

Shimp and Sharma (1987) associate the concept of ethnocentrism with the identification of the ingroups, which are those groups with which an individual identifies with, while the outgroups are the groups with which the individual does not identify with. In this way, aspects of the personality of the individual are related to his or her social and cultural frame of reference. In functional terms, ethnocentrism then gives the individual a factor of identity through a feeling of belonging; for this reason, his or her behaviors are oriented and judged by his or her group of origin (Witkowski, 1998). For Caruana (1996), ethnocentrism from a sociological perspective has a social function of strengthening cohesion and solidarity among the members of a group, but it can also contribute toward an attitude of superiority, intolerance, and even contempt for those who have different customs. Taking this into account, ethnocentrism then refers to the inability of an individual to perceive reality from any other point of view than that of his or her own culture or social group, and, for that reason, he or she judges other groups from that personal reference (Thomas and Hill, 1999).

However, some previous research has shown how an ethnocentric society can have favorable feelings toward foreign brands in specific situations. For Wang and Chen (2004), in developing countries, it can be seen how imported products represent a symbolic meaning that generates social status and a social distinction. For Ger and Belk (1999), conspicuous consumers tend to show their wealth by buying imported products. In this way, the preference to buy domestic products and reject foreign products does not always occur, especially if foreign products are perceived as symbols that allow them to stand out socially, and are associated with a higher quality, image, and prestige (Wang and Chen, 2004).

On this point, Karoui and Khemakhem (2019) also consider that consumers in developing countries could accept the purchase of imported goods if these generate some kind of social recognition. In this way, consumers can express ethnocentric trends but, at the same time, conspicuous consumption trends, where, in this case, there may be inclined by their conspicuous trends. In their study, Kavak and Gumusluoglu (2007) concluded that, in groups with a clear family orientation, although there are ethnocentric values in some members of the family, these will not totally oppose the purchase of foreign products, provided that these foreign products enable the family to improve its social status. On the other hand, for Areiza-Padilla et al. (2020), in emerging markets, it is possible that a group can be ethnocentric and, at the same time, consume foreign brands; however, these foreign brands must be very strong and have values of globality, modernity, prestige, and success. From this, the following hypothesis was put forward.

#### Hypothesis 2

Ethnocentrism has a positive impact on the conspicuous consumption of global brands that practice sustainability in developing countries.

### Patriotism

Patriotism refers to the positive perceptions that a person has toward his or her own country of origin and, therefore, toward his or her compatriots, customs, and national identity (Durvasula and Lysonski, 2008). However, although this concept includes pride in the country of origin of an individual, it is different from the concept of nationalism. Nationalism refers to the vision that people have, believing that their country of origin is superior to other countries, generating a totally uncritical feeling; however, patriotic feelings are related only to internal qualities and do not criticize other countries (Sinkkonen, 2013). Ishii (2009) considers that patriotism is the love that a citizen has for his or her country of origin, generating feelings of attachment and loyalty toward this country, but without generating hostility toward foreign countries. We can say that the difference between patriotism and nationalism lies in attitudes toward other countries. In this way, patriotism is compatible with attitudes that foster internationalization and cooperation among countries, while nationalism correlates negatively with these values. In this way, patriotism can be compatible with tolerance and economic and cultural diversity, while nationalism is associated with values of authoritarianism and intolerance (Li and Brewer, 2004).

For Eng and Bogaert (2010), conspicuous consumption represents a way to show the wealth and success of some citizens in a given society. In this way, these consumers become a social reference for the rest of the citizens, who are in a lower social hierarchy. This situation is more common in developing countries where the upper classes tend to have conspicuous consumption, which ends up being an example to be followed by the lower classes. In this way, if the upper classes consume products that they consider patriotic and represent symbols of national identity, the lower classes will also consider these same products as patriotic and as symbols of national identity. This is because consumers in the lower classes often repeat the behaviors of the upper classes as a mechanism that allows them to aspire toward that social group in a higher hierarchy. In other words, they generate false upward social mobility (González, 2017; Costa Filho et al., 2021).

On the other hand, in the study of Areiza-Padilla et al. (2020), it can be observed how companies of foreign origin that offered conspicuous consumption can manage to highlight the patriotism of their local consumers when they managed to diminish the perception of being a foreign brand. This is possible by symbolically nationalizing the brand by highlighting the symbols of the national identity of that local country. This is possible through different strategies. For example, when it is possible to stand out in the decoration of commercial premises, various symbols of national identity represent the country of that consumer, in addition to offering typical products of that country.

For example, the Starbucks chain, as a global and foreign brand in an emerging market, developed its strategy of penetration into the Colombian market by exaltation of the national identity symbols of that country. For this, their premises were adapted and customized for this country. Its decoration was based on showing the landscapes alluding to Colombia through the use of images that highlight the Colombian coffee grower, the main landscapes of the regions where coffee is planted in Colombia, and all kinds of audiovisual strategy related to the production, sale, and consumption of Colombian coffee. On the other hand, in its communication strategy to the Colombian public, it was guaranteed that Starbucks in Colombia would only offer 100% coffee in its liquid coffee to the Colombian consumers, highlighting its commitment to the Colombian coffee grower and to this country. On the other hand, among the products offered at Starbucks, there is a variety of typical products from Colombia that are only available in that country; in this way, the Colombian consumers can feel like any coffee shop of Colombian origin. In this way, Colombian coffee is highlighted as a symbol of national identity for this country through a foreign brand (Areiza-Padilla et al., 2020).

Taking into account the above and based on previous studies by Lu Wang and Chen (2004), Eng and Bogaert (2010), Cervellon and Shammas (2013), González (2017), Semaan et al. (2019), Spielmann et al. (2020), and Costa Filho et al. (2021), for this investigation, we considered that the consumption of certain products or brands that generate conspicuous consumption in the upper classes will produce a purchasing behavior imitated by the lower classes. Thus, if the upper classes consider that the products they buy generate a feeling of patriotism, the lower classes will also consider that the consumption of these products exalts national identity and patriotism. In this way, a positive relationship between patriotism and conspicuous consumption can be observed, so we formulate the following hypothesis:

#### Hypothesis 3

Patriotism has a positive impact on the conspicuous consumption of global brands that practice sustainability in developing countries.

### Brand Image

Brand image is directly related to the perception of a consumer of a specific brand and both its functional and symbolic benefits; even, in some situations, the consumer can value its symbolic benefits more than the functional benefits of the product (Nandan, 2005). In this way, brand image refers to the thoughts and feelings that the consumer has about a particular brand. These characteristics are fundamental for any company, since they allow it to develop a competitive advantage both rationally and emotionally, which allows them to be differentiated from their direct competitors (Roy and Banerjee, 2007).

In this way, if the brand has positioning in the mind of the consumer through values such as commercial superiority, quality, and prestige; these values will end up influencing the purchase decision of the consumer to choose this brand over other brands (Hsieh and Li, 2008). In this way, the more symbolic and positive characteristics the brand represents in a given society, the stronger the positive feelings toward its brand image. In this way, if a brand generates social status in that society, the desire to consume it by the members of that specific society will increase (O'Cass and Frost, 2002).

For these reasons, we can say that the brand image can be positively related to conspicuous consumption, as these consumers seek to improve their social status through displaying a symbolic consumption, and certain brands allow them to represent these values of luxury and prestige. In this way, some consumers have a clear preference for the purchase and use of certain specific brands, which have previously been positioned as prestige and social reputation brands. Therefore, these types of consumers will be willing to pay higher prices, compared with other brands that do not have the same social weight (O'Cass and Frost, 2002).

In this way, there can be a strong symbolic relationship between the image of a brand and the self-concept of a consumer, if the consumer can reflect on his or her lifestyle, his or her values, and his or her social and economic aspirations through a specific brand. In this case, such a brand should then be able to represent its set of symbolic, emotional, and social beliefs (O'Cass and Frost, 2002). Taking this into account, we can explain the preference that the conspicuous consumer has for the purchase and use of certain brands, whose image is previously positioned in the market, as brands generate social status (Assimos et al., 2019; Li et al., 2019). In this way, we formulate the following hypothesis:

#### Hypothesis 4

Conspicuous consumption has a positive impact on the brand image of global brands that practices sustainability in developing countries.

### Brand Loyalty

Brand loyalty generates an emotional bond in the consumer through the attributes and values represented by the brand; this makes consumers choose a particular brand over other brands, even at lower prices or with similar characteristics (Ogba and Tan, 2009). In this way, brand loyalty can generate benefits for both its customers and brand owners. For customers, emotional and symbolic benefits are generated, as the brand represents the same values and attitudes with which the consumer feels identified and can represent his or her lifestyle.

On the other hand, the brand generates economic benefits for the owners of the brand, as customers often repeat on several occasions the purchase of that brand and also usually recommend it to third parties, which, following these recommendations, end up buying that brand also (Bowen and Chen, 2001). On the other hand, brand loyalty can become a strong barrier to entry for new competitors because of its positioning in the minds of consumers, who, in most cases, are unwilling to change brands. In this way, brand loyalty allows the company to respond to the threats of the competition and generate greater sales by having customers less sensitive to the price. In this way, brand loyalty is considered one of the ways in which the consumer expresses his or her satisfaction with the performance of the product or service received (Delgado-Ballester and Munuera-AlemaÂn, 2001).

For Chaudhuri and Holbrook (2001), brand loyalty occurs when a consumer has a clear preference for buying a specific brand above other brands regardless of their value or their functional characteristics. For this reason, loyalty to the brand influences both the attitudes and the behavior of the consumer since he or she feels an emotional commitment that forces him or her to buy this brand and to speak well of it (Chai et al., 2015).

Taking into account the symbolic value represented by the brands, a positive connection is generated with the symbolic value represented by conspicuous consumption. In this way, brands that symbolize a social status will allow the conspicuous consumer to exhibit the values and attributes associated with that social status. For this reason, the conspicuous consumer previously identifies the brands that generate that social distinction and generates loyalty to those brands (Ehrenberg and Goodhardt, 1970).

With this in mind, and based on previous studies by O'Cass and Frost (2002), Amaldoss and Jain (2005), and Jacob et al. (2020), for this research, we consider that conspicuous consumers are loyal to certain brands that allow them to satisfy not only their material needs but also their symbolic needs of prestige, luxury, and social distinction, which is the most evident in emerging markets, where there is strong pressure to excel socially. With this in mind, we propose the following hypothesis:

#### Hypothesis 5

Conspicuous consumption has a positive impact on brand loyalty that is global and practices sustainability in developing countries.

On the other hand, the brand image is positively related to the purchasing intention of the consumer, and, in the same way, it positively influences customer satisfaction, which, in the end, allows to generate brand loyalty toward it (Huang et al., 2020). In this way, the brand image ends up positively influencing brand loyalty, meaning that when a consumer has a very positive brand image, his or her brand loyalty to it will also be greater (Greve, 2014). According to Wu (2011), brand image has direct effects on brand loyalty, generating a tendency to repeat the purchase. For Sondoh et al. (2007), when customers perceive greater social benefits through a brand, they tend to be more loyal to that brand. In this way, there is a symbolic and affective value, which ends up being an indicator of purchase and repurchase. For this reason, they consider that there is a relationship between the functionality, the prestige of the brand, with the expression of the personality of the consumer, which seeks to have a similarity with the values and attitudes of a specific brand. In this way, for Bauer et al. (2008), a strategy that marketing specialists could develop is to boost consumer preferences and brand loyalty through the development of strong, positive, and unique beliefs about consumers, which is through the construction of a solid brand image. With this in mind, we propose the following hypothesis:

#### Hypothesis 6

Brand image has a positive impact on brand loyalty that is global and practices sustainability in developing countries.

### Research Model

**Figure 1:**
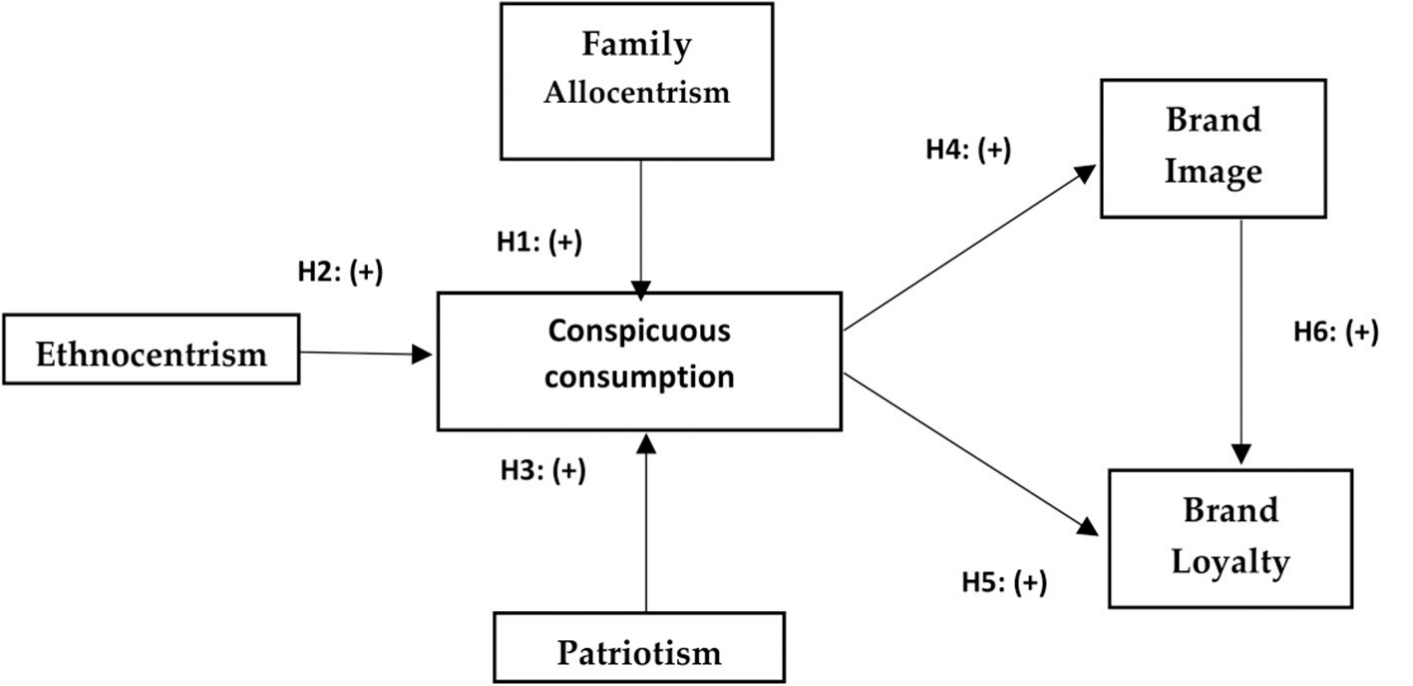
Research model and hypothesis.

Figure 1 shows the model of this research with their respective hypotheses.

## Methodology

For this research, we have focused on the American coffee chain Starbucks as a global brand and on Colombia as a developing country. For some authors, such as Lin (2012), Smith Maguire and Hu, 2013, and Wu et al. (2019), Starbucks clearly generates conspicuous consumption in emerging markets due to the values associated with its brand, such as its globality, in addition to its North American origin. This origin of the brand is associated with a more developed, more modern country with a greater economy, and, therefore, a country that is aspirational for some consumers in these emerging countries. In the case of the Colombian market, it should be noted that Colombia is a country with a very culturally and economically rooted coffee culture, since it is the third country exporting coffee in the world.

Due to the massification of its domestic consumption, coffee is a product that is obtained abundantly in the Colombian market at very cheap prices. By the date of this study, a cup of coffee in a popular sector is priced at approximately $.15 US dollars; however, this same cup of coffee can cost in Starbucks $1.15 US dollars; that is seven times more than in a popular sector. Because Starbucks presents various types of products, such as cappuccino and frappuccino, among other products, their prices can rise above $2.50. Although, in Colombia, the direct competitor of Starbucks is “Juan Valdez,” Starbucks still has the highest prices in the whole country regarding this type of coffee shops.

Starbucks entered the Colombian market in 2014; its first store was located in one of the most exclusive sectors of the Colombian capital to initially attract the wealthiest people in the city. In this way, their consumption allows to generate social notoriety, because not all can pay their high prices. At the time of this study, Starbucks has 23 stores throughout the country, always in very socially recognized places and with very attractive shops, which is why it has positioned itself in the Colombian market as one of the most expensive cafes in the country.

It is important to mention that one of the entry strategies of Starbucks in the Colombian market was a communication strategy where they informed the Colombian consumers that Starbucks, being a sustainable company, pays a “fair price” to Colombian coffee growers so that they have a better quality of life. In this way, Starbucks in Colombia pays a higher price for the coffee bags than the usual price in the market. This way, the coffee growers who sell their crops to Starbucks receive more income. On the other hand, Starbucks trains and promotes the responsible and fair production of the Colombian peasant and favors the production of organic products. For this reason, it monitors the social and environmental impacts of its suppliers, such as waste management, water protection, conservation of natural resources, preservation of biodiversity, and reduction of the use of agrochemicals, etc. (Redacción el Tiempo, 2015).

For this reason, Colombia has one of the “coffee grower support centers” in the city of Manizales, which owns Starbucks in various countries around the world, with the aim of supporting Colombian producers regardless of whether or not they sell their coffee to Starbucks, providing tools that allow them to reduce production costs and the management and control of pests and diseases that affect the quality of coffee. Currently, in Colombia, more than 60,000 farms in 12 departments of the country participate in this specific program. It also encourages the use of reusable containers and recycling processes for its waste in its stores. These actions have allowed it to stand out and position itself as a company with social and economic responsibility within the Colombian market (Starbucks Stories and News, 2016).

In view of the above, for this research, a quantitative study was conducted through a structured questionnaire to customers over 18 years of age, the Starbucks coffee chain in the city of Bogotá, Colombia. All the participants were previously told that the survey was anonymous, that their personal data would not be requested, and that the results would be processed globally and not individually. Similarly, the survey was voluntary and could be discontinued at any time. The data were collected in mid-2019, obtaining a final sample of 305 valid questionnaires.

The data were processed through the technique partial least squares (PLS-SEM). For this research, we decided to use this technique, because it allows us to handle both formative and reflective variables in the same study (Diamantopoulos and Winklhofer, 2001). For this study, the brand image variable is formative according to the author of the scale we chose (Palacios-Florencio et al., 2018). Similarly, the PSL allows a better adaptation to predictive studies compared with other tools (Barroso et al., 2010). The results were analyzed in two stages. In the first stage, an exploratory factor analysis was conducted, using the SPSS program, to analyze the possible dimensions of conspicuous consumption and ethnocentrism variables, while, in the second stage, a confirmatory factor analysis was carried out to validate the measuring instrument, and the structural model was estimated, using partial least squares (PLS), using the Smart PLS 3.2.7 software.

Scales previously validated in the literature in previous studies were adapted for this study. The seven-point Likert scale was used as follows: 1 = “totally disagree” and 7 = “totally agree.” The variable of conspicuous consumption was measured using the scale of Marcoux et al. (1997). This scale uses 5 dimensions, which are described below: materialistic hedonism, communication of belonging, social status demonstration, interpersonal mediation, and ostentation. For family allocentrism, the four items of Triandis and Gelfand (1998), alluding to the family in the vertical collectivism resignation, were used. For ethnocentrism, the 24 items on the scale of Sharma (2015) were used, while, for patriotism, the nine items of Levinson (1950) were used. The nine items proposed by Palacios-Florencio et al. (2018) were used to measure the brand image, and, finally, the three items proposed by Kim et al. (2008) were used to measure brand loyalty.

Regarding the demographic profile of the participants, the sample had a very equitable gender distribution, where 51.8% were men, and 48.2% were women. Regarding age, 81% were in the age range of 18 and 35 years, 17.4% between 36 and 55 years, and only 1.6% were older than 56 years. Of this sample, the vast majority of 57% had undergraduate or postgraduate university studies, and 43.3% worked as employees, while 38.4% were still studying.

## Analysis and Discussion of the Results

### Exploratory Factor Analysis

To determine the dimensionality of the variable conspicuous consumption and ethnocentrism in order to determine which items should measure each dimension and if the items were grouped in the way in which it was initially proposed, an exploratory factor analysis was conducted with VARIMAX rotation. Thus, first, it was found that the items used to measure the conspicuous consumption construct were grouped into five factors, which, according to the semantic content of the items collected in each of them, have been named: materialistic hedonism, communication of belonging to/dissociation from a group, social status demonstration, interpersonal mediation, and ostentation, jointly explaining 89% of the variability of the phenomenon, providing a satisfactory adjustment. Replicating the exploratory analysis on the scale used to measure ethnocentrism, 24 items were retained, from which three factors emerged that have been called: affective reaction, cognitive bias, and behavioral preference, and which together account for 83% of the phenomenon, providing a satisfactory adjustment. Table 2 shows the results of the KMO (Kaiser-Meyer-Olkin) sample adequacy measurement index and the Bartlett sphericity test for the corresponding analyses.

**Table 2:** Summary of exploratory factor analysis.

**Construct**	**KMO**	**Bartlett's test (Chi-square value)**	**Significance**	**Dimensions**
Conspicuous consumption	0.922	3,657.57	0.000	5
Ethnocentrism	0.945	5,289.76	0.000	3

*Source: Author's own compilation*.

### Confirmatory Factor Analysis

For the evaluation of the confirmatory factor analysis, in the measurement models composed by reflective items (Mode A), the reliability of the first order constructs was analyzed, the individual reliability of the item (α of Cronbach), and the measure of composite reliability was analyzed (CR). With respect to convergent validity, all indicator loads were significant and >0.7 (except for one item of the family allocentrism variable and two items of the patriotism variable that were eliminated). In addition, the mean extracted variance (AVE) value of each variable was >0.5, providing evidence of adequate convergent validity in the measurement model (Fornell and Larcker, 1981).

Table 3 shows the results of the AFC. For the brand image variable, when defined as formative (Mode B), its evaluation is done at the level of the indicators by assessing the possible multicolineality through the variance inflation factor (VIF) and the assessment of the magnitude of their weights and their significance, the results of which can be seen in Table 4. Regarding the discriminant validity, for the verification of its compliance, the Fornell and Larcker criterion and the Heterotrait-Heteromethod-HT and Mono-trait-Heteromethod-MT (HTMT) ratio were used.

**Table 3:** Measurement model evaluation results.

**Construct/Indicators**	**Mean**	**St.dev**.	**Loadings factor**
**F1. Family allocentrism (α = 0.803; CR = 0.791; AVE = 0.573)**			
Parents and children must stay together as much as possible.	5.38	1.47	0.751^*^
It is my duty to take care of my family, even when 1 have to sacrifice what I want.	5.44	1.43	0.757^*^
It is my duty to take care of my family, even when 1 have to sacrifice what I want.	4.97	1.63	0.992^*^
**F2. Patriotism (α = 0.842; CR = 0.881; AVE = 0.517)**			
There will always be superior and inferior nations in the world and, in the interests of all concerned, it is best that the superior ones be in control of world affairs.	3.71	1.83	0.764^*^
Minor forms of military training, obedience and discipline, such as drill, marching and simple commands, should be made a part of the elementary school educational program.	3.63	1.84	0.747^*^
The main threat to basic Colombian institutions during this century has come from the infiltration of foreign ideas, doctrines and agitators.	3.72	1.91	0.737^*^
Present treatment of conscientious objectors, draft evaders and enemy aliens is too lenient and mollycoddling. If a person won't fight for his country, he deserves a lot worse than just prison or a work camp.	3.20	1.92	0.792^*^
In view of the present national emergency, it is highly important to limit responsible government jobs to native, white, Christian Colombians.	2.89	1.82	0.750^*^
Foreigners refugees may need them, but it would be a big mistake to lower your immigration quotas and allow them to flood the country.	3.84	1.97	0.737^*^
Colombia can never advance to the standards of living and civilization of the U.S., due mainly to the innate dirtiness, laziness and general backwardness of Colombian.	3.89	1.89	0.786^*^
**F3. Ethnocentrism-Affectivereaction (α = 0.946; CR = 0.955; AVE = 0.726)**			
I love the services from Colombia.	4.77	1.39	0.753^*^
I am proud of the services from Colombia.	4.54	1.52	0.872^*^
I admire the services from Colombia.	4.51	1.47	0.844^*^
I feel attached to the services from Colombia	4.36	1.53	0.868^*^
I hate the services from foreign countries.	4.19	1.59	0.892^*^
I despise the services from foreign countries.	4.73	1.51	0.851^*^
I am embarrassed by the services from foreign countries.	4.44	1.69	0.879^*^
I feel no attachment with the services from foreign countries.	4.18	1.72	0.849^*^
**F4. Ethnocentrism-Cognitivebias (α = 0.886; CR = 0.909; AVE = 0.562)**			
East or West, the services from Colombia are the best.	3.97	1.59	0.780^*^
Services from Colombia are examples of best workmanship.	4.19	1.66	0.802^*^
Service providers from Colombia have the best work attitudes.	4.34	1.49	0.737^*^
Products and services from foreign countries are no match for those from Colombia	3.53	1.72	0.755^*^
Colombia has the hardest working people in the services sector.	4.66	1.49	0.705^*^
Service providers from Colombia are more caring than those in any foreign country	3.99	1.44	0.810^*^
Services from Colombia are guaranteed for best performance.	4.11	1.37	0.845^*^
Colombia provides the most pleasant service experience.	4.31	1.66	0.801^*^
**F5. Ethnocentrism-Behavioralpreference(α = 0.889; CR = 0.909; AVE = 0.556)**			
For me, it's always the services from Colombia first, last and foremost.	4.15	1.71	0.770^*^
If I have a choice, I would prefer buying services from Colombia.	4.69	1.69	0.782^*^
I prefer being served by service providers from Colombia.	4.73	1.53	0.711^*^
As far as possible, I avoid buying services from foreign countries.	4.71	1.58	0.745^*^
I often refuse to buy service because it is from a foreign country.	3.53	1.65	0.743^*^
I would much rather not buy a product or service than buy one from a foreign country.	3.53	1.67	0.741^*^
It may cost me in the long run but I support services from Colombia.	3.95	1.71	0.799^*^
I will never regret buying a service from Colombia.	4.14	1.85	0.766^*^
**F6. Conspicuous consumption-Materialistic hedonism (α = 0.856; CR = 0.895; AVE = 0.631)**			
People buy foreign services to enhance their image.	5.04	1.73	0.794^*^
People buy foreign services for uniqueness, to have services others do not own.	4.83	1.71	0.776^*^
People buy foreign services to be fashionable.	5.36	1.58	0.806^*^
By using foreign services people intend to please others.	4.64	1.69	0.802^*^
People using foreign services feel more important.	5.18	1.61	0.880^*^
**F7. Conspicuous consumption-Communication of belonging to/dissociation from a group (α = 0.831; CR = 0.889; AVE = 0.675)**			
People want to have foreign services owned by their friends and colleagues.	3.98	1.80	0.910^*^
People want to have foreign services owned by their neighbors.	3.73	1.87	0.916^*^
People want foreign services owned by everybody.	3.61	1.87	0.867^*^
People buy foreign services to show off to be noted.	5.13	1.66	0.730^*^
**F8. Conspicuous consumption-Social status demonstration (α = 0.834; CR = 0.888; AVE = 0.729)**			
Services from foreign are social status symbols.	4.94	1.79	0.709^*^
Services from foreign are a symbol of success and prestige.	4.53	1.79	0.909^*^
Services from foreign mean wealth.	4.17	1.89	0.934^*^
**F9. Conspicuous consumption-Interpersonal mediation (α = 0.885; CR = 0.921; AVE = 0.744)**			
People using foreign services increase their own value from the point of view of others.	4.06	1.99	0.856^*^
People using foreign services are more attractive than others	3.38	2.00	0.867^*^
Use of foreign services allows popularity among friends and colleagues.	4.04	1.91	0.857^*^
Using foreign services induces respect from others.	3.29	1.89	0.869^*^
**F10. Conspicuous consumption-Ostentation (α = 0.786; CR = 0.903; AVE = 0.823)**			
If people could afford it only foreign services would be bought	3.63	1.94	0.923^*^
People buy foreign services only because they are more expensive than Polish products.	3.65	1.90	0.891^*^
**F11. Brand Loyalty (α = 0.914; CR = 0.946; AVE = 0.853)**			
I consider myself to be loyal to this café chain.	3.23	1.90	0.927^*^
The café chain would be my first choice.	3.31	1.99	0.924^*^
I am very likely to switch to another café chain brand that runs promotions.	3.46	1.94	0.919^*^

*Source: Own compilation of the author, α, Cronbach's Alpha; CR, composite reliability; AVE, average variance extracted;*

* *p < 0.01*.

**Table 4:** Measurement construct brand image.

**Indicators**	**Mean**	**St.dev**.	**Weights**	**VIF**	***t***	***p*-value**
The location of café chain is suitable	5.32	1.39	−0.233	1.429	2.777	0.006
I can clearly distinguish the establishments of this café chain	5.38	1.53	−0.116	1.390	2.376	0.018
I tend to pay attention to the information they send me	4.33	1.74	0.132	1.364	1.988	0.047
This café chain's image fits my personality	4.43	1.71	0.166	1.446	2.243	0.025

*Source: Author's own compilation*.

As shown in Table 5, on the one hand, it is evident that the estimated correlation between two factors is less than the square root of the variance-extracted average of each factor (Fornell and Larcker, 1981), and, on the other hand, the values for the HTMT ratio are <0.9 Henseler et al. (2015), thus confirming the discriminating validity of the reflective structures of the measurement model. Regarding the second-order reflective constructs, Table 6 shows the standardized loads, which are >0.7 and statistically significant for all dimensions, the Cronbach α, the composite reliability measurement (CR), and the extracted variance analysis (AVE), and it is evident that first-order dimensions contribute statistically significantly to their corresponding second-order reflective constructs.

**Table 5:** Discriminant validity.

	**F1**	**F2**	**F3**	**F4**	**F5**	**F6**	**F7**	**F8**	**F9**	**F10**	**F11**	**F12**
F1	**0.757**	0.312	0.336	0.254	0.346	0.104	0.087	0.091	0.117	0.098	0.131	NA
F2	0.334	**0.719**	0.265	0.301	0.349	0.251	0.411	0.344	0.509	0.469	0.469	NA
F3	0.224	0.239	**0.852**	0.731	0.638	0.225	0.340	0.203	0.283	0.254	0.196	NA
F4	0.204	0.256	0.684	**0.750**	0.761	0.175	0.325	0.160	0.273	0.292	0.263	NA
F5	0.287	0.338	0.585	0.682	**0.745**	0.284	0.417	0.183	0.252	0.315	0.236	NA
F6	0.052	0.214	0.216	0.146	0.240	**0.794**	0.733	0.714	0.498	0.510	0.125	NA
F7	0.104	0.345	0.307	0.300	0.369	0.568	**0.822**	0.646	0.690	0.683	0.243	NA
F8	0.108	0.334	0.191	0.151	0.170	0.561	0.501	**0.854**	0.792	0.597	0.197	NA
F9	0.127	0.444	0.266	0.260	0.256	0.454	0.596	0.703	**0.862**	0.823	0.478	NA
F10	0.126	0.390	0.229	0.243	0.293	0.435	0.555	0.506	0.695	**0,907**	0.438	NA
F11	0.193	0.419	0.186	0.232	0.239	0.049	0.227	0.218	0.433	0.373	**0.923**	NA
F12	0.177	0.275	0.093	0.122	0.146	0.087	0.255	0.238	0.423	0.293	0.633	NA

*Source: Own compilation of the author. On the diagonal: square root of the AVE values. Below the diagonal: correlations. Above the diagonal: HTMT values; NA, not applicable. F12 = image*.

*The values on the diagonal in bold represent the square root of the variance extracted. The values below the diagonal correspond to the estimated correlation between factors*.

**Table 6:** The measurement model of the second-order construct.

		**Loadings dimension**	**α**	**CR**	**AVE**
Ethnocentrism	SET-Affective reaction	0.859^*^	0.848	0.908	0.767
	SET-Cognitive bias	0.893^*^			
	SET-Behavioral preference	0.874^*^			
Conspicuous consumption	CC-Materialistic hedonism	0.766^*^	0.863	0.898	0.641
	CC-Communication of belonging to/dissociation from a group	0.792^*^			
	CC-Social status demonstration	0.797^*^			
	CC-Interpersonal mediation	0.901^*^			
	CC-Ostentation	0.829^*^			

*Source: Own compilation of the author, α, Cronbach's alpha; CR, composite reliability; AVE, average variance extracted;*

* *p < 0.01*.

### The Structural Model and Hypotheses Testing

After the validation of the measuring instrument, the estimation of the structural model was carried out, using the PLS technique and the Henseler (2017) bootstrapping procedure, with 5,000 subsamples. As can be seen in Table 7, the coefficients of the paths have been significant in all cases and, in the sense, indicated by the hypotheses, except in the relationship between family allocentrism and conspicuous consumption.

**Table 7:** Structural model results.

**Hypothesis and Relationship**	**Original Sample**	***t***	***p*-value**	**Contrast**
H1: Family allocentrism—Conspicuous consumption	0.059	0.613	0.540	Not accepted
H2: Ethnocentrism—Conspicuous consumption	0.241	4.168	0.000	Accepted
H3: Patriotism—Conspicuous consumption	0.393	6.760	0.000	Accepted
H4: Conspicuous consumption—Brand image	0.358	7.100	0.000	Accepted
H5: Conspicuous consumption—Brand loyalty	0.159	3.103	0.002	Accepted
H6: Brand image—Brand loyalty	0.578	12.691	0.000	Accepted

*Source: Own compilation of the author. R^2^ (conspicuous consumption) = 0.253; R^2^ (image) = 0.128; R^2^ (loyalty) = 0.426; Q^2^ (conspicuous consumption) = 0.153; Q^2^ (image) = 0.046; Q^2^ (loyalty) = 0.356*.

The importance-performance map (IPMA) analysis, also known as the “priority map,” provides a valuable additional analysis of the results obtained in the evaluation of the structural model, which contrasts the total effects (or importance) of the structural model and average score values of latent variables (or performance) to identify significant areas of improvement where business efforts should be concentrated (Hair et al., 2016), especially when these key spaces are of relatively high importance for the target variable but have a relatively low performance (Ringle and Sarstedt, 2016). The target variable in our case is brand loyalty. Based on the importance and performance averages, the map has been divided into four quadrants in which the latent variables are located according to their importance and performance.

**Figure 2:**
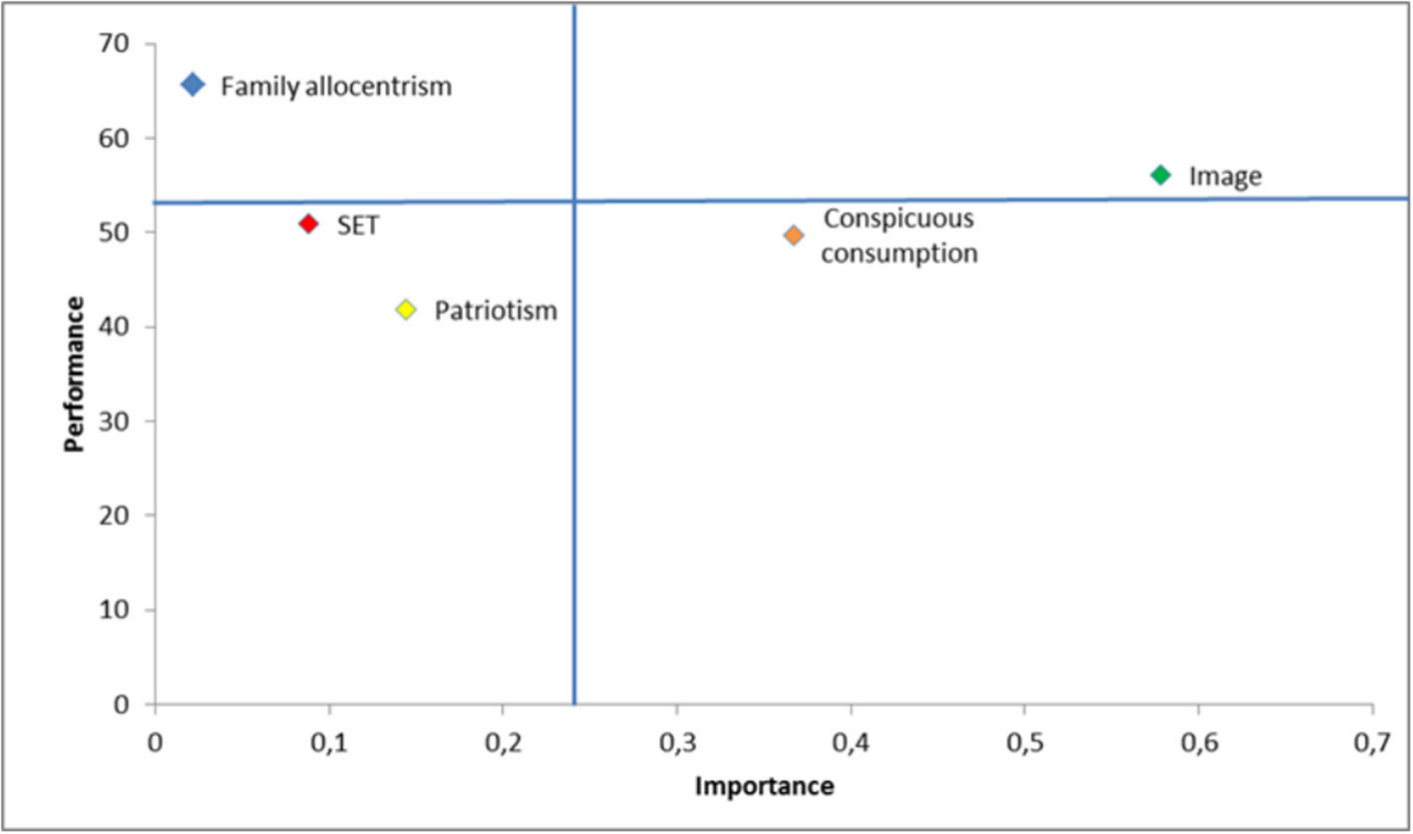
Importance-performance map of the target construct loyalty. Source: Author's own compilation.

The results provide managers with a guide for prioritizing business decision-making, with the aim of improving the different areas of interest in their organizations. According to Ringle and Sarstedt (2016), when analyzing the significance-performance map, constructs located in the lower right area (i.e., above-average importance and below-average performance) are of greater interest in achieving improvement, followed by those in the upper-right area, lower-left area, and, finally, upper-left area. As can be seen in Figure 2, looking at the lower-right area of the importance-performance map, it is observed that the conspicuous consumption has a considerably greater importance (0.367) than the other variables, except the brand image.

These findings are in line with previous studies in this field such as those by Podoshen and Andrzejewski (2012); therefore, when managers aim to increase brand loyalty performance, their first priority should be to improve customer conspicuous consumption performance, as this construct has high (above average) importance, but a low (below average) performance. Second, with a total effect of 0.578, the importance of the brand image is particularly high. Thus, a one-unit increase in brand image performance would increase brand loyalty performance by 0.578 points (*ceteris paribus*). Third, our findings have revealed that ethnocentrism and patriotism have lower yields (51,979 and 41,879) and also below-average levels of importance (0.088 and 0.144), meaning that there is a great deal of room for improvement in these areas. Finally, the variable family allocentrism, by not having a significant effect on brand loyalty, should not capture the attention of managers in the organizations, since it is located in the lower business priority quadrant.

## Discussion, Research Implications, and Limitations

### Discussion and Theoretical Implications

This study has attempted to contribute to the scarce literature that exists between the possible positive relationship between conspicuous consumption and global brands that carry out sustainable practices in emerging markets. In this way, contributions are generated that will be of great value to the managers of global and conspicuous brands, who want their brands to contribute to economic, social, and environmental sustainability, both for the company and for society, in general.

The results show that there is no significant relationship between family allocentrism and conspicuous consumption. Initially, a positive and significant relationship between the two variables was proposed in the hypothesis (H1), but the results show that the relationship between the two is not significant. For this reason, we had to reject this hypothesis. This may be because conspicuous consumption is more common in individualistic societies than in collectivist societies, as individualists tend to have higher materialistic values in order to achieve their own individual goals, where their individual development is framed in a strong competition in search of personal success, where personal achievements are valued more than the social relationships developed by a person (Wong, 1997; Arai and Pedlar, 2003).

Secondly, we were able to verify the positive and significant relationship between patriotism and conspicuous consumption, confirming previous studies by Eng and Bogaert (2010) and Semaan et al. (2019); thus, it can be concluded that patriotism in emerging countries like Colombia encourages conspicuous consumption, when these products or services are seen as part of that of the symbols of the national identity of that country.

Third, this study verifies the positive and significant relationship between consumer ethnocentrism in developing countries, with conspicuous consumption, based on previous studies by Karoui and Khemakhem (2019), Kavak and Gumusluoglu (2007), and Mai and Tambyah (2011); in this way, it can be observed that people in emerging markets, although they may be ethnocentric, when the purchase of foreign products generates social status, and, in addition, these products are part of the symbols of national identity, negative feelings toward them are not generated.

Fourth, this study confirms the positive and significant relationships between conspicuous consumption, brand image, and brand loyalty according to previous studies by Assimos et al. (2019), Li et al. (2019), Podoshen and Andrzejewski, 2012, and Palumbo and Herbig, 2000; in this way, we can say that conspicuous consumers usually have a very positive brand image of the brands they buy and that they generate this social status, and, for this reason, they have a brand loyalty toward them.

Finally, this research confirms the positive and significant relationship between brand image and brand loyalty, as did previous studies by Huang et al. (2020) and Bauer et al. (2008); in this way, we can say that the brand image is a precedent of brand loyalty.

### Managerial Implications

This study has several implications for marketing managers who want their global and conspicuous brands to be able to generate sustainability strategies without losing the social status they generate for their consumers. We can conclude that conspicuous consumption should not always be negatively related to sustainability. In some cases, their consumption can even help popularize sustainable consumption. In the case of Starbucks as a global brand, in emerging markets, its consumption is considered conspicuous (Lin, 2012; Smith Maguire and Hu, 2013; Wu et al., 2019). In the specific case of Colombia, the price of its products is seven times higher than that of the products of the popular sectors, in addition because its strategy of entering the Colombian market was to position itself in this way in the mind of the consumer. In Colombia, Starbucks generates in its consumers notoriety that allows it to stand out socially, and that is why its customers are willing to pay higher prices than their close competitors. In this way, the values of globalization, social status, and economic success that the brand has are passed on to the people who buy it. On the other hand, Starbucks in Colombia continues in its communication and promotion strategy, frequently remembering that they are a sustainable brand.

They constantly state that the price they pay to the Colombian coffee growers is a “fair price,” and, for this reason, they pay a higher price for each sack of coffee, at the price established in the market by the National Federation of Coffee Growers of Colombia; that is to say that, thanks to them, Colombian coffee growers have better incomes and, therefore, a better quality of life.

They also often recall how they help the Colombian coffee growers in the training of environmentally friendly practices for coffee production, in addition to offering organic products, and encourage the use of reusable containers and waste recycling processes in their shops. On the other hand, through their support center for coffee growers in the city of Manizales, they support all Colombian producers who want it regardless of whether or not they sell their coffee to Starbucks, providing them with tools that allow them to reduce production costs and control the pests of their crops.

In this way, they have managed to position themselves in the mind of the consumers as a brand with sustainable processes that help society and the environment, but also continue to preserve their values of exclusivity and social status. In this way, the consumption of Starbucks turns out to be both sustainable and conspicuous consumption, since the people who buy their products contribute indirectly to social, environmental, and economic sustainability, and, on the other hand, being a global and expensive brand, it generates ostentatious consumption that allows to excel socially.

## Limitations and Future Research

To conclude, we propose some limitations of this study, which, at the same time, suggest future lines of research. The first is the geographical limitation of the sample, where we propose to analyze emerging markets from different continents in order to know if the cultural characteristics of each region influence the results. Similarly, researchers can replicate this study in developed countries to identify if the development of a country changes the perception of its consumers in these issues.

Second, ethnocentrism, family allocentrism, and patriotism in this study were antecedents of conspicuous consumption, so it would be interesting to analyze the direct relationship of these constructs to brand image and brand loyalty of global brands that practice sustainability in developing countries. Thirdly, this study was conducted with a multinational hedonic services company, and it would be interesting to know whether similar results would be presented with utilitarian goods.

Qualitative techniques could also be used to make views of consumers known directly and their adaptability to other industries. Finally, new variables could be included in the model such as the experience of consumption and the perception of consumers toward global brands after the COVID pandemic to see if, after the economic problems, the perception of it remains the same.

## Data Availability Statement

The original contributions presented in the study are included in the article/supplementary material, further inquiries can be directed to the corresponding author/s.

## Author Contributions

JA-P: conceptualization, methodology, investigation, data curation, visualization, supervision, and project administration. JA-P and MM: software, validation, formal analysis, writing—original draft preparation, and writing—review and editing. Both authors have read and agreed to the published version of the manuscript.

## Conflict of Interest

The authors declare that the research was conducted in the absence of any commercial or financial relationships that could be construed as a potential conflict of interest.

## Publisher's Note

All claims expressed in this article are solely those of the authors and do not necessarily represent those of their affiliated organizations, or those of the publisher, the editors and the reviewers. Any product that may be evaluated in this article, or claim that may be made by its manufacturer, is not guaranteed or endorsed by the publisher.
